# Deep Learning Method Based on Multivariate Variational Mode Decomposition for Classification of Epileptic Signals

**DOI:** 10.3390/brainsci15090933

**Published:** 2025-08-27

**Authors:** Shang Zhang, Guangda Liu, Shiqing Sun, Jing Cai

**Affiliations:** College of Instrumentation and Electrical Engineering, Jilin University, Changchun 130061, China; zhangshang20@mails.jlu.edu.cn (S.Z.); sqsun23@mails.jlu.edu.cn (S.S.); caijing1979@jlu.edu.cn (J.C.)

**Keywords:** multivariate variational mode decomposition, deep learning, electroencephalogram, focal epileptic signal classification, multi-class seizure type classification

## Abstract

**Background/Objectives**: Epilepsy is a neurological disorder that severely impacts patients’ quality of life. In clinical practice, specific pharmacological and surgical interventions are tailored to distinct seizure types. The identification of the epileptogenic zone enables the implementation of surgical procedures and neuromodulation therapies. Consequently, accurate classification of seizure types and precise determination of focal epileptic signals are critical to provide clinicians with essential diagnostic insights for optimizing therapeutic strategies. Traditional machine learning approaches are constrained in their efficacy due to limited capability in autonomously extracting features. **Methods**: This study proposes a novel deep learning framework integrating temporal and spatial information extraction to address this limitation. Multivariate variational mode decomposition (MVMD) is employed to maintain inter-channel mode alignment during the decomposition of multi-channel epileptic signals, ensuring the synchronization of time–frequency characteristics across channels and effectively mitigating mode mixing and mode mismatch issues. **Results**: The Bern–Barcelona database is employed to classify focal epileptic signals, with the proposed framework achieving an accuracy of 98.85%, a sensitivity of 98.75%, and a specificity of 98.95%. For multi-class seizure type classification, the TUSZ database is utilized. Subject-dependent experiments yield an accuracy of 96.17% with a weighted F1-score of 0.962. Meanwhile, subject-independent experiments attain an accuracy of 87.97% and a weighted F1-score of 0.884. **Conclusions**: The proposed framework effectively integrates temporal and spatial domain information derived from multi-channel epileptic signals, thereby significantly enhancing the algorithm’s classification performance. The performance on unseen patients demonstrates robust generalization capability, indicating the potential clinical applicability in assisting neurologists with epileptic signal classification.

## 1. Introduction

Epilepsy is characterized as a neurological disorder marked by recurrent seizures, which originate from excessive electrical discharges in brain cells, leading to abnormal cortical activity [1]. Recurrent seizure episodes can lead to physical injuries and neuropsychological impairments, subsequently contributing to mental disorders such as anxiety and depression. In severe cases, sudden epileptic seizures may result in traumatic injuries, including fractures and contusions, with potential life-threatening consequences [2,3]. According to epidemiological statistics, this condition affects approximately 50 million people worldwide, while up to 70% of patients could live seizure-free through proper diagnosis and appropriate therapeutic interventions [4].

Epileptogenic zones are identified as the origin areas of epileptic seizures and represent the critical cortical regions that need to be precisely localized during surgical treatment and can potentially induce seizure activities [5]. These regions may not necessarily correspond to structurally visible lesions observable through imaging techniques, such as tumors and cortical dysplasia, and their identification requires a comprehensive assessment based on electroencephalography and clinical manifestations [6]. Surgical resection or complete disconnection of the epileptogenic zones is considered the most critical step toward achieving long-term seizure-free status in patients with epilepsy. Theoretically, the complete removal of these regions can eliminate epileptic symptoms. In clinical practice, the identification of focal epileptic signals can lead to the location of electrode channels with focal signals, enabling precise localization of epileptogenic zones, and thereby facilitating surgical resection or neuromodulation therapies [7,8]. Epileptic seizures can be classified based on the onset origin, propagation extent, symptom manifestations (e.g., motor symptoms, degree of consciousness impairment), and etiological characteristics [9]. The differences in the treatment of various seizure types are primarily reflected in etiological targeting, medication selection, intervention methods, and prognosis. To ensure effective treatment and minimize adverse effects, the seizure type must first be correctly identified, as some targeted medications are only applicable to specific conditions. In the worst cases, due to the mechanism of action of the medication, its use in patients with other seizure types may even exacerbate symptoms [10]. Furthermore, the classification of seizure types provides critical guidance for clinicians to formulate tailored treatment strategies, including antiepileptic drug regimens or surgical planning [9,11]. Therefore, accurately classifying epileptic signals constitutes an essential research component in clinical epilepsy treatment.

Electroencephalogram (EEG) signals are generated by spontaneous electrical discharges of cerebral neurons, containing substantial physiological and pathological information reflecting intracerebral activities [12]. EEG plays a critical role in epilepsy diagnosis and treatment. EEG is utilized to distinguish epileptic seizures from non-epileptic seizures (e.g., syncope, motor disorders, migraine) to prevent misdiagnosis, while simultaneously localizing cortical regions of seizure propagation for precise identification of epileptogenic zones [13]. EEG measurements are divided into two modalities: scalp EEG, which records the spontaneous electrical activity of neuronal populations through electrodes placed on the scalp surface [12], and intracranial EEG (iEEG), involving intracranial electrode implantation through navigation-assisted or surgical procedures to capture neuronal electrical signals [14]. Clinical classification of epileptic signals currently relies on qualified experts to perform visual analysis of lengthy EEG recordings, which is a time-consuming and labor-intensive process, particularly during long-term monitoring lasting several days [7]. Moreover, diagnostic accuracy in epilepsy diagnosis remains heavily dependent on the individual clinician’s expertise and experience, potentially leading to misdiagnosis due to subjective interpretation. These limitations highlight the urgent clinical necessity to design and develop effective epileptic signal classification methodologies.

Signal decomposition refers to the mathematical process of representing a complex signal as the sum of several simple signal components, facilitating analysis, feature extraction, and subsequent processing. It can extract the intrinsic characteristics of signals, optimize signal processing efficiency, and support engineering application requirements. It is extensively applied in various fields such as signal processing, communications, and data analysis. Dragomiretskiy et al. proposed variational mode decomposition (VMD), a multiscale time–frequency signal analysis method based on variational optimization [15]. The fundamental principle involves decomposing complex signals into multiple intrinsic mode functions (IMFs) with specific center frequencies and limited bandwidth, which exhibit spectral separation and local orthogonality in the frequency domain. The objective of VMD is to achieve adaptive frequency–domain decomposition by solving a constrained variational problem. The synchronous optimization process in VMD enables the one-time extraction of all mode components, thereby circumventing the error accumulation issues associated with conventional recursive decomposition methods such as empirical mode decomposition (EMD). Furthermore, by constraining the minimization of each mode’s bandwidth, VMD effectively separates harmonic components with adjacent frequencies, making it particularly suitable for precise analysis of multi-component non-stationary signals. Notably, VMD is specifically designed for univariate signal decomposition. To address this limitation, Rehman et al. proposed multivariate variational mode decomposition (MVMD) [16]. MVMD improves noise resistance ability through multivariate joint modeling during decomposition. This multivariate extension enables simultaneous processing of multi-channel data while maintaining mode alignment across channels, ensuring synchronized time–frequency characteristics among decomposed components. Such capability effectively mitigates mode mixing and phase misalignment issues caused by independent single-channel decomposition, rendering MVMD particularly advantageous for analyzing multi-channel EEG signals.

The current methodologies for epileptic signal classification predominantly employ machine learning techniques. Although discernible characteristic differences exist in different types of epileptic EEG signals, the inherent strong non-stationary and nonlinear characteristics of EEG signals significantly compromise classification performance [17]. Manual feature engineering refers to the process of manually designing and constructing essential features capable of effectively representing raw data, relying on the knowledge and experience of domain experts. The primary objective is to preserve crucial information while eliminating redundant data. This approach is heavily dependent on human expertise and requires redesigning for different datasets, resulting in low efficiency and poor generalization capabilities. Deep learning approaches circumvent these limitations by automatically extracting complex feature representations from raw data through multilayer neural networks, eliminating reliance on manually engineered feature extraction [18]. Furthermore, the hierarchical architecture of deep learning models enhances nonlinear representation capabilities and complex pattern recognition capacities, rendering them particularly suitable for processing nonlinear biological signals. As a result, the application of deep learning frameworks for epileptic signal classification has emerged as a new research trend.

Considering the characteristic properties of epileptic EEG signals, this study proposes a novel deep learning framework for epileptic signal classification. Owing to the informational constraints inherent in single-channel EEG signals, the proposed method employs MVMD adapted for multi-channel EEG signals to decompose signals into IMFs. A convolutional neural network (CNN) is implemented to extract local spatial features contained within spectrograms, followed by a bidirectional gated recurrent unit (BiGRU) and a Transformer network extracting local and global temporal characteristics, respectively, thereby comprehensively characterizing epileptic signals through the effective integration of temporal and spatial domain features. The integration of residual networks significantly alleviates the issues of gradient vanishing and explosion while accelerating training convergence. The classification performance of the proposed framework is evaluated using two public databases. The main contributions of the paper can be summarized as follows:

• This study employs MVMD to decompose multi-channel epileptic EEG signals, thereby generating mode-aligned IMF components across channels. This approach effectively mitigates mode mixing and mode mismatch issues, thereby facilitating enhanced feature extraction by deep learning networks.
• A deep learning framework is implemented to jointly capture local spatial and temporal features from epileptic EEG signals, achieving effective epileptic signal classification. Gradient vanishing and explosion phenomena are mitigated through the integration of residual networks, which optimize model stability and enable the learning of complex mapping relationships.
• The proposed framework is validated using two public databases for two distinct classification tasks: focal epileptic signal classification and multi-class seizure type classification. The superior classification performance substantiates the framework’s strong generalization capability. For multi-class seizure type classification, the model’s performance is comprehensively evaluated under both subject-dependent and subject-independent experiments. The superior performance achieved on unseen patients highlights its strong generalization ability and significant potential for clinical application.

The subsequent sections of this paper are structured as follows. Section 2 introduces the related works. Section 3 details the proposed MVMD and the deep learning framework. Section 4 shows the experimental setup and results. Section 5 presents the discussions and comparisons of results. Finally, the conclusions are given in Section 6.

## 2. Related Work

### 2.1. Related Works in Classifying Focal Epileptic Signals

The research on focal epileptic signal classification is dedicated to the development of advanced algorithms for the high-accuracy identification of focal epileptic signals to localize epileptogenic zones in patients. To achieve this objective, numerous algorithms have been applied to focal epileptic signal classification. During this period, machine learning methods have been extensively employed to address these challenges [19,20,21,22]. Murariu et al. extracted spectral power densities of IMFs through EMD, followed by the application of k-nearest neighbor (KNN) and naive Bayes (NB) classifiers for distinguishing focal and non-focal signals [23]. The classifiers achieved satisfactory classification accuracy, demonstrating a potential methodology for focal epileptic signal classification. Akbari et al. implemented empirical wavelet transform (EWT) to decompose epileptic signals into multiple rhythms, from which nonlinear features were subsequently extracted [24]. The support vector machine (SVM) and KNN classifiers were ultimately employed. The algorithm exhibited robust discrimination capabilities for small and large datasets, providing valuable insights for epileptogenic zone localization. Abhishek et al. systematically investigated the classification performance of two fractal parameters combined with power spectral densities and spectral entropy [25]. Experimental results indicated that the random forest (RF) and extra tree classifiers demonstrated optimal performance.

The classification of focal epileptic signals using deep learning methods has attracted increasing attention in recent research. Srinath et al. employed EMD for feature extraction, followed by classification through a CNN, achieving superior accuracy [26]. This study successfully distinguished focal signals and further classified them as mild or severe cases, thereby providing enhanced guidance for clinicians in evaluating patient conditions. Wang et al. introduced a multi-branch deep learning fusion framework [27]. This methodology demonstrated effective identification of epileptogenic zones, presenting a potential diagnostic tool. Modak et al. implemented cross wavelet transform for feature extraction prior to CNN-based classification, achieving peak performance in delta rhythm and offering a viable solution for epileptogenic zone localization [28]. The limitations of current research predominantly focus on the binary classification task for focal epileptic signal classification, while critical gaps remain in validating algorithmic performance on multi-class classification tasks. In particular, insufficient attention has been devoted to investigating algorithm generalizability across diverse clinical requirements.

### 2.2. Related Works in Classifying Multi-Class Seizure Types

Early investigations into multi-class seizure type classification were predominantly focused on machine learning approaches [29]. In seizure type classification based on machine learning approaches, feature extraction relies heavily on manual engineering based on domain expertise, followed by rigorous feature selection to reduce computational complexity. Albaqami et al. implemented the dual-tree complex wavelet transform (DTCWT) combined with statistical features to achieve classification through LightGBM [30]. This methodology reported weighted F1-scores of 0.991 and 0.747 for seizure-wise and patient-wise classification, respectively.

Although machine learning approaches have demonstrated remarkable success in seizure type classification, these methods heavily rely on manual feature engineering and dimensionality reduction to balance classification performance with computational complexity. In this context, deep learning methods have been increasingly adopted to integrate multi-domain information for seizure type classification [31,32,33,34]. Li et al. proposed a channel-embedding spectral–temporal squeeze-and-excitation network (CE-stSENet), which simultaneously incorporated spectral and temporal domain information [35]. Priyasad et al. introduced an attention-driven data fusion architecture, providing a methodological framework for classifying seizure types [34]. Regarding the limitations of multi-class seizure type classification, most existing algorithms are exclusively validated on an individual database, lacking a comprehensive assessment of algorithmic generalizability across multiple databases. Multi-database validation is crucial for evaluating the capability to process signals with varying distributions and verifying effectiveness across diverse tasks, thereby enhancing the adaptability of algorithms in clinical applications.

The excessive training duration and number of parameters associated with complex deep learning architectures have motivated researchers to investigate signal decomposition and feature extraction methodologies integrated with deep learning approaches for seizure type classification. Tuncer et al. employed discrete wavelet transform (DWT) and a correlation-based feature selection for feature extraction and selection, followed by the implementation of long short-term memory (LSTM) to classify seizure types in 23 subjects, achieving satisfactory classification accuracy [36]. However, the investigation was confined to two seizure types: CPSZ and ABSZ. Shankar et al. proposed a hybrid model combining Hilbert vibration decomposition with continuous wavelet transform (CWT), subsequently processed through a CNN and LSTM [37]. This framework demonstrated favorable classification performance in extracting spatial and temporal features across 153 seizures from 30 patients. Nevertheless, the study was constrained by the limited number of seizures and patients analyzed. Sarić et al. implemented CWT coupled with an artificial neural network for epileptic diagnosis in 315 patients, obtaining promising classification accuracy [38]. The methodology was successfully deployed on hardware, demonstrating potential for clinical applications. The limitations of current approaches predominantly neglect critical subject-independent considerations. As clinical deployment necessitates algorithms to process EEG signals on unseen patients, rigorous evaluation of model generalizability and classification efficacy on unseen subjects is imperative.

Compared with other studies, this paper proposes a model combining the MVMD decomposition method with a deep learning framework, achieving effective decomposition of multi-channel epileptic signals and enhancing mode alignment across channels. Subsequently, the deep learning framework is utilized to effectively extract local spatial features and temporal characteristics, significantly improving classification performance. This innovative approach is not only applied to the classification of focal epileptic signals but also the classification of multi-class seizure types, enabling validations through multiple databases and diverse tasks. This reduces the shortcomings of insufficient testing caused by other studies that use an individual database for single-task operation, effectively enhancing our algorithm’s generalization across databases and tasks. This paper conducts subject-dependent and subject-independent experiments for the classification of multi-class seizure types, addressing the deficiency of algorithm analysis on unseen subjects and effectively enhancing our algorithm’s generalization across diverse patients.

## 3. Materials and Methods

### 3.1. Epileptic EEG Databases

The first database is the Bern–Barcelona database obtained from the Department of Neurology at the University of Bern [39]. This database contains two categories of iEEG recordings, encompassing focal signals and non-focal signals, from five patients diagnosed with pharmacoresistant temporal lobe epilepsy. The signals were recorded with intracranial strip and depth electrodes. Signals initially recorded from the epileptogenic zones were labeled as focal signals, whereas other signals were labeled as non-focal signals. Based on the localization of the epileptogenic zones, surgical resection could be performed on the seizure onset zone of the brain. The channels where the first ictal EEG signal was detected were regarded as the focal EEG channels, while the remaining channels were regarded as non-focal EEG channels. Each category comprises 3750 record files and a total recording duration of 75,000 s, with each record file containing a signal pair. For focal signals, the signal pair was derived from focal EEG channels, with one focal channel being signal x and its neighboring focal channel being signal y. Conversely, for non-focal signals, the signal pair was derived from non-focal EEG channels. To ensure parameter consistency, this paper utilizes the signal pairs of full duration sampled at 512 Hz for each category, ultimately leading to a binary classification task. This database is suitable for focal epileptic signal classification.

The second database is the Temple University Hospital EEG Seizure Corpus (TUSZ) [40]. This database contains clinically recorded scalp EEG signals encompassing eight distinct seizure types, comprising 3050 seizure events from 306 patients. The seizure types include generalized non-specific seizure (GNSZ), focal non-specific seizure (FNSZ), absence seizure (ABSZ), simple partial seizure (SPSZ), complex partial seizure (CPSZ), tonic seizure (TNSZ), tonic clonic seizure (TCSZ), and myoclonic seizure (MYSZ). To maintain consistent data parameters, patient records with a sampling rate of 256 Hz from the 01_tcp_ar and 03_tcp_ar_a folders were utilized, and the selected records contained only one seizure type. The ABSZ and MYSZ types were excluded due to non-compliance with these screening criteria, resulting in the final classification of six remaining seizure types. The detailed information of the database is shown in Table 1. The bipolar Temporal Central Parasagittal average reference EEG montage was adopted as the electrode standardization protocol. To optimize computational complexity while maintaining classification performance, the number of electrode channels was minimized to seven: FP1, F4, C3, P4, O1, F7, and T6. The database is suitable for multi-class seizure type classification.

### 3.2. The Proposed Approach

**Figure 1:**
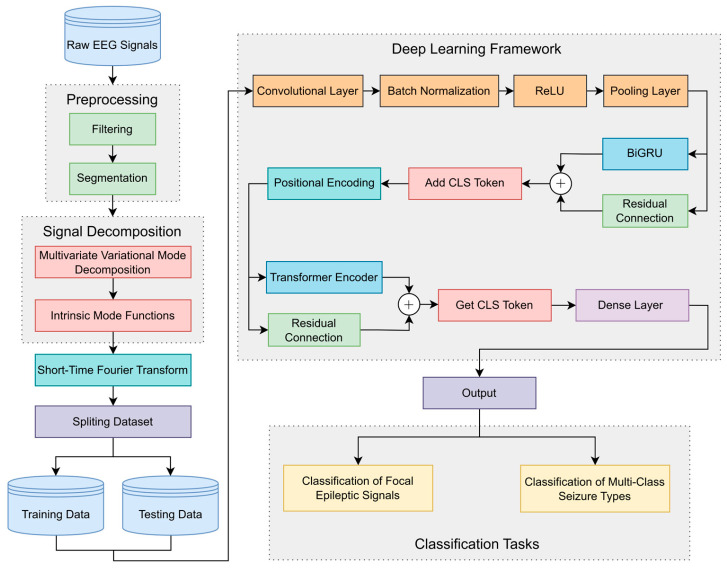
The framework of the proposed architecture.

The proposed approach integrates signal decomposition with a deep learning framework to effectively extract feature information from epileptic signals and achieve classification tasks. The framework of the proposed architecture is illustrated in Figure 1.

Initially, raw EEG signals undergo preprocessing comprising data filtering and segmentation. Specific frequency bands to be retained are extracted through data filtering to eliminate random signal fluctuations, thereby enhancing data reliability and stability. Data segmentation partitions the EEG signals into smaller segments, facilitating feature extraction and preventing excessive computational load.

Subsequently, MVMD is employed for channel joint decomposition, generating a predetermined number of IMF components. Each IMF component represents a modulated oscillation at a specific frequency scale, preserving the local characteristics and energy distribution of the original data. The decomposition maintains inter-channel coupling relationships, ensuring frequency–domain alignment of same-order modes and preserving cross-channel correlations. Following this, short-time Fourier transform (STFT) is applied to each IMF component across all channels to obtain spectrograms containing transient spectral–temporal information. The processed data are divided into training and testing data according to labels. The spectrograms are then fed into the deep learning framework.

The data sequentially pass through a CNN composed of a convolutional layer, batch normalization, ReLU, and a pooling layer to capture local spatial information from spectrograms. The data flow is subsequently directed into a BiGRU network to extract local temporal features and capture short-distance dependency relationships. Inspired by Vision Transformer, a CLS Token is incorporated into the data flow via the add CLS Token module. This token participates in feature extraction as a unified sequence element, interacting with other features to acquire global information. Positional encoding is then integrated to enable positional awareness. The data flow is processed by the Transformer encoder module to obtain global temporal features and long-distance dependency relationships. Residual connections are implemented for BiGRU and Transformer networks, where cross-layer skip pathways enhance the network’s capacity to learn complex mappings and optimize model stability. Finally, the CLS Token is retrieved as the information representation vector, which is classified using a dense layer. For focal epileptic signal classification, the data are categorized into focal and non-focal classes. In multi-class seizure type classification, the data are classified into six seizure types.

#### 3.2.1. Multivariate Variational Mode Decomposition

MVMD is introduced to process multivariate data with an arbitrary number of channels. The primary objective lies in extracting modal components with common frequency characteristics from multivariate data, particularly addressing multi-source signal processing requirements in biomedical engineering and industrial monitoring domains. MVMD enhances mode alignment capability through multi-channel collaborative optimization, effectively mitigating cross-channel noise interference and improving decomposition consistency.

MVMD is employed to extract K predefined IMF components, $u_{k}(t)$, with common frequency components from input data $x(t)=[x_{1}(t),x_{2}(t),\ldots x_{C}(t)]$ containing C channels:

(1) $$x(t)=\sum_{k=1}^{K}\;u_{k}(t)$$

where $u_{k}(t)=[u_{k,1}(t),u_{k,2}(t),\ldots u_{k,C}(t)]$.

MVMD is required to adhere to the criterion that the summation of extracted modes accurately reconstructs the original signal while simultaneously minimizing the total bandwidth of decomposed modes. The analytic signal of $u_{k}(t)$, represented by $u_{+}^{k}(t)$, is derived via the Hilbert transform. The specific construction procedure is outlined as follows: (1) The analytic signal of each IMF component is computed to obtain the unilateral frequency spectrum; (2) the unilateral frequency spectrum is shifted to the baseband by multiplying with $e^{-j\omega_{k}t}$, where $\omega_{k}$ represents the center frequency component; and (3) $H^{1}$ Gaussian smoothness is utilized to estimate the bandwidth of modes. The constrained optimization problem for MVMD can be formulated as follows:

(2) $$\left\{\begin{array}{c} \underset{\left\{u_{k,c}\},\{\omega_{k}\right\}}{minimize}\;\left\{\sum_{k}\sum_{c}{∥\partial_{t}\left[u_{+}^{k,c}\left(t\right)e^{-j\omega_{k}t}\right]∥}_{2}^{2}\right\}\\ \mathrm{subject}\;\mathrm{to}\;\sum_{k}u_{k,c}\left(t\right)=x_{c}\left(t\right),c=1,2,\ldots ,C \end{array}\right.$$

where $u_{+}^{k,c}$ is the analytic signal corresponding to channel c and mode k.

The corresponding augmented Lagrangian function is formulated as

(3) $$\begin{array}{cc} L\left(\left\{u_{k,c}\right\},\left\{\omega_{k}\right\},\lambda_{c}\right) & \\ & =\alpha \sum_{k}\;\sum_{c}\;{∥\partial_{t}\left[u_{+}^{k,c}\left(t\right)e^{-j\omega_{k}t}\right]∥}_{2}^{2}+\sum_{c}\;{∥x_{c}\left(t\right)-\sum_{k}\;u_{k,c}\left(t\right)∥}_{2}^{2}\\ & +\sum_{c}\;\left\langle \lambda_{c}\left(t\right),x_{c}\left(t\right)-\sum_{k}\;u_{k,c}\left(t\right)\right\rangle \end{array}$$

where α represents the penalty coefficient, which ensures reconstruction accuracy under Gaussian noise contamination. $\lambda_{c}$ denotes the Lagrange multiplier for channel c, maintaining the strictness of constraint conditions.

The solution to the original optimization problem can be obtained by identifying the saddle point of the Lagrangian function. The complex optimization problem can be decomposed into a sequence of iterative sub-optimization problems using the alternate direction method of multipliers (ADMM). The iteration formulas are expressed as

(4) $$u_{k,c}^{n+1}\leftarrow \underset{u_{k,c}}{arg\;min}\;L(\{u_{i<k,c}^{n+1}\},\{u_{i\geq k,c}^{n}\},\{\omega_{i}^{n}\},\lambda_{c}^{n})$$

(5) $$\omega_{k}^{n+1}\leftarrow \underset{\omega_{k}}{arg\;min}\;L(\{u_{i,c}^{n+1}\},\{\omega_{i<k}^{n+1}\},\{\omega_{i\geq k}^{n}\},\lambda_{c}^{n})$$

(6) $$\lambda_{c}^{n+1}=\lambda_{c}^{n}+\tau \left(x_{c}-\sum_{k}\;u_{k,c}^{n+1}\right)$$

The convergence criterion is described as

(7) $$\sum_{k}\;\sum_{c}\;\frac{∥u_{k,c}^{n+1}-u_{k,c}^{n}{∥}_{2}^{2}}{∥u_{k,c}^{n}{∥}_{2}^{2}}<\epsilon$$

where τ indicates the Lagrangian multiplier update parameter and ϵ indicates the convergence tolerance.

To derive the mode update expression, Equation (4) is reformulated as the following equivalent form:

(8) $$u_{k,c}^{n+1}=\underset{u_{k,c}}{arg\;min}\;\left\{\alpha {∥\partial_{t}\left[u_{+}^{k,c}(t)e^{-j\omega_{k}t}\right]∥}_{2}^{2}+{∥x_{c}(t)-\sum_{i}\;u_{i,c}(t)+\frac{\lambda_{c}(t)}{2}∥}_{2}^{2}\right\}$$

The minimization problem is resolved in the spectral domain, yielding the frequency–domain mode update:

(9) $${\hat{u}}_{k,c}^{n+1}(\omega )=\frac{{\hat{x}}_{c}(\omega )-\sum_{i\neq k}\;{\hat{u}}_{i,c}(\omega )+\frac{{\hat{\lambda }}_{c}(\omega )}{2}}{1+2\alpha (\omega -\omega_{k}{)}^{2}}$$

Subsequently, the update expression for the central frequency is derived. As only one term in Equation (3) depends on $\omega_{k}$, Equation (5) is rewritten as

(10) $$\omega_{k}^{n+1}=\underset{\omega_{k}}{arg\;min}\;\left\{\sum_{c}\;{∥\partial_{t}\left[u_{+}^{k,c}(t)e^{-j\omega_{k}t}\right]∥}_{2}^{2}\right\}$$

Through application of the Plancherel theorem, the optimization problem is transformed into the frequency domain, leading to the equivalent formulation:

(11) $$\omega_{k}^{n+1}=\underset{\omega_{k}}{arg\;min}\;\left\{\sum_{c}\;\int_{0}^{\infty }\;(\omega -\omega_{k}{)}^{2}|{\hat{u}}_{k,c}(\omega ){|}^{2}d\omega \right\}$$

The extreme value of Equation (11) provides the solution to the central frequency optimization problem, resulting in the center frequency update:

(12) $$\omega_{k}^{n+1}=\frac{\sum_{c}\;\int_{0}^{\infty }\;\omega |{\hat{u}}_{k,c}(\omega ){|}^{2}d\omega }{\sum_{c}\;\int_{0}^{\infty }\;|{\hat{u}}_{k,c}(\omega ){|}^{2}d\omega }$$

The frequency bands of decomposed signals are obtained through update relationships, generating k IMF components. Notably, MVMD allows for simultaneous processing of multi-channel data, ensuring inter-channel frequency consistency and enhancing analytical stability.

#### 3.2.2. CNN

CNNs, which are specifically designed for processing multidimensional grid data, are predominantly employed in image classification, object detection, image segmentation, and medical image analysis. A CNN is generally composed of convolutional layers and pooling layers.

The convolutional layers, which primarily comprise convolution operations and activation functions, are responsible for extracting hierarchical feature representations from raw input data. The convolution kernel, serving as the core component of convolution operations, is implemented as a small-scale weight matrix for local feature extraction. The convolution kernel performs dot product operations with the input local region through a sliding window to calculate weighted sums, thereby capturing specific patterns and generating feature maps. Convolution operations can generate many feature maps using different convolution kernels, reflecting different aspects of input information. The activation function maps the weighted sum of inputs to a nonlinear space. Common activation functions include sigmoid, tanh, and ReLU. Sigmoid and tanh functions suffer from gradient vanishing issues during training. In this study, ReLU is utilized to alleviate this problem.

The pooling layer is primarily employed for dimensionality reduction, thereby diminishing computational load and the number of learnable parameters. This operation executes down-sampling through a sliding window on input feature maps and preserves salient features within local receptive fields. The predominant methods currently utilized include max pooling and average pooling. Max pooling extracts the maximum element from the filter-covered region of the feature map to preserve prominent feature representations, whereas average pooling computes the mean value to smooth feature distributions. In this study, AdaptiveAvgPool is adopted, which automatically adjusts both the pooling window size and stride based on predefined output dimensions. This operation partitions the input feature map and calculates the average value for each partition, ensuring strict matching of the output feature maps. This operation maintains feature consistency and effectively reduces model parameter quantity and model complexity while preserving critical feature information.

#### 3.2.3. BiGRU

Gated recurrent unit (GRU) is an enhanced recurrent neural network (RNN) architecture designed to mitigate gradient vanishing and explosion problems inherent in conventional RNNs through a gating mechanism composed of an update gate and a reset gate. The GRU unit controls information flow through update and reset gates, offering higher computational and parameter efficiency compared to LSTM.

The update gate, which is responsible for determining the proportion of new information to be updated and old information to be retained, is mathematically formulated as

(13) $$z_{t}=\sigma \left(W_{z}\cdot \left[h_{t-1},x_{t}\right]\right)$$

where $z_{t}$ denotes the output of the update gate, $h_{t-1}$ represents the hidden state for the previous time step, $x_{t}$ indicates the current input, $W_{z}$ corresponds to the weight parameter of the update gate, and σ signifies the sigmoid activation function that constrains output values within the interval [0, 1].

The reset gate controls the influence of historical information on the current candidate state. When reset gate values approach zero, nearly all historical information is discarded, which is particularly applicable in scenarios where forgetting certain information is required. Conversely, when reset gate values are close to one, the historical information is nearly entirely preserved. The formula is expressed as

(14) $$r_{t}=\sigma (W_{r}\cdot [h_{t-1},x_{t}])$$

where $r_{t}$ denotes the output of the reset gate, and $W_{r}$ denotes the weight parameter of the reset gate.

The candidate hidden state is generated by integrating the current input with the adjusted historical information, which is calculated as

(15) $$\tilde{h_{t}}=tanh\;(W_{h}\cdot [r_{t}*h_{t-1},x_{t}])$$

where $\tilde{h_{t}}$ denotes the candidate hidden state, which serves as the temporary memory at the current time step. $W_{h}$ represents the weight parameter of the candidate hidden state. The function tanh constrains output values within the interval [−1,1], thereby guaranteeing stable updates of the hidden states.

The final hidden state update $h_{t}$ integrates the previous state and the candidate state through the update gate. The larger the update gate, the greater the contribution of the candidate state to the new hidden state, indicating that the network incorporates more new information from the current time step. Conversely, the smaller the update gate, the more historical information is retained, enabling the network to preserve its previous memory. The formula is represented as

(16) $$h_{t}=[(1-z_{t})*h_{t-1}]+[z_{t}*\tilde{h_{t}}]$$

**Figure 2:**
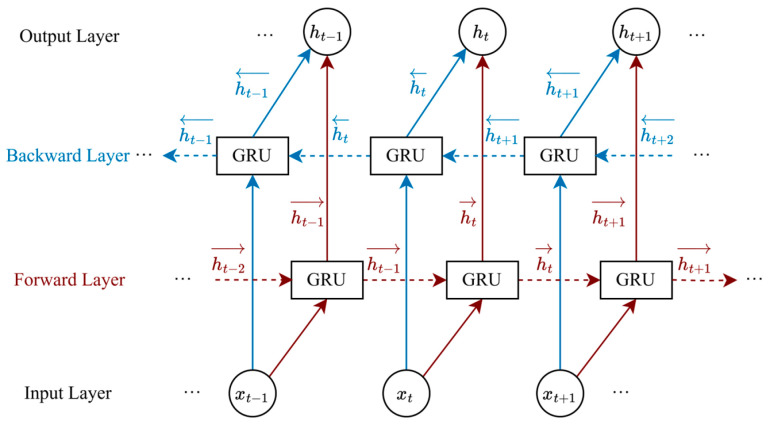
The diagram of the BiGRU structure.

BiGRU is a deep learning model that integrates a bidirectional architecture with GRU units, and is extensively employed in sequence classification and natural language processing tasks. A diagram of the BiGRU structure is shown in Figure 2. BiGRU comprises two GRU layers, one forward and one backward, which capture information from the past and future directions of the sequence, respectively, enhancing bidirectional modeling capabilities. The update formula is expressed as

(17) $$\left\{\begin{array}{c} \vec{h_{t}}=GRU(x_{t},\vec{h_{t-1}})\\ \overset{\leftarrow }{h_{t}}=GRU(x_{t},\overset{\leftarrow }{h_{t+1}})\\ h_{t}=[\vec{h_{t}},\overset{\leftarrow }{h_{t}}] \end{array}\right.$$

where $\vec{h_{t}}$ and $\overset{\leftarrow }{h_{t}}$ denote the hidden states of the forward and backward GRU, respectively. $h_{t}$ represents the output of BiGRU.

#### 3.2.4. Transformer

Transformer is fundamentally designed by self-attention mechanisms and encoder-decoder structures, demonstrating remarkable capabilities in natural language processing and multimodal tasks. This architecture is constructed through the stacked encoder and decoder layers. The encoder processes input sequences through multi-head self-attention layers and feed-forward networks (FFNs), while the decoder employs masked self-attention layers to facilitate sequential generation.

The self-attention mechanism dynamically captures long-distance dependencies by computing association weights between arbitrary positions within input sequences. This mechanism permits direct interactions between arbitrary positions, effectively overcoming limitations imposed by local receptive fields. Multi-head self-attention layer captures global dependencies at different positions through multiple attention heads, generating feature representations with dynamically weighted allocations. FFNs process the features of each position independently and enhance nonlinear expression ability through fully connected layers with nonlinear activation functions. Since the self-attention mechanisms lack sequence order information, positional features need to be injected through positional encoding. Positional encoding assigns a unique encoding to each position in sequences, thereby compensating for the sequence-insensitive nature of self-attention mechanisms.

## 4. Results

### 4.1. Experimental Setup

**Figure 3:**
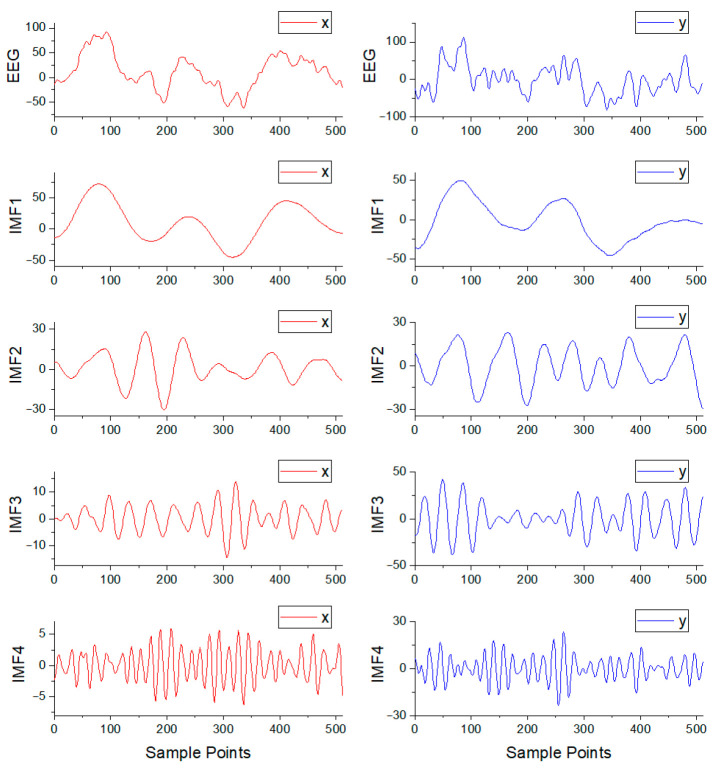
The focal signal pair for one epoch from the Bern–Barcelona database and its corresponding four IMF components.

The data from the Bern–Barcelona database were processed through 50 Hz notch filtering to eliminate power-line interference and 0.5–50 Hz band-pass filtering to eliminate high-frequency interference. The signals were segmented into one-second epochs, with the MVMD algorithm subsequently applied to each epoch. For each category, 10,000 epochs were randomly selected for subsequent analysis. This paper involves many hyperparameters, and using the grid search method will lead to high computational costs and low efficiency. Therefore, this study employed the random search method to optimize hyperparameters and obtained suitable hyperparameter results. The decomposition hyperparameters were configured as follows: α is 2000, and K is 4. The STFT implementation hyperparameters were as follows: window is Hann, nperseg is 128, and overlap is 64. Figure 3 illustrates a focal signal pair for one epoch and its corresponding four IMF components.

**Figure 4:**
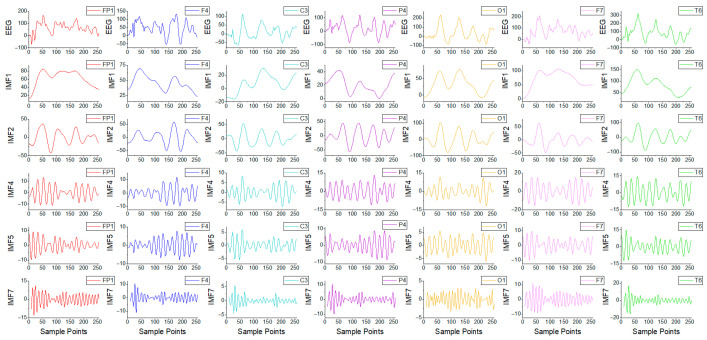
The GNSZ signal for one epoch with 7 channels from the TUSZ database and its partial IMF components.

The TUSZ dataset was subjected to 60 Hz notch filtering to eliminate power-line interference and 0.5–50 Hz band-pass filtering to eliminate high-frequency interference. Recordings were also segmented into one-second epochs. For each seizure type, 2000 epochs were randomly selected. In cases of SPSZ, TNSZ, and TCSZ types with insufficient seizure duration, data augmentation was implemented through the method of sliding window overlapping sampling to alleviate class imbalance issues. The hyperparameter results were obtained through a random search method. The decomposition hyperparameters were configured as follows: α is 2000, and K is 8. The STFT implementation hyperparameters were as follows: window is Hann, nperseg is 64, and overlap is 32. Figure 4 illustrates the GNSZ signal for one epoch with seven channels and partial IMF components.

The deep learning architecture was trained using the Adam optimizer and the StepLR scheduler with a batch size of 64, epochs of 200, an initial learning rate of 0.001, a step size of 5, gamma of 0.1, and the loss function of CrossEntropyLoss. After training the model, the detailed data dimensions of each module can be obtained to understand the model construction. For the Bern–Barcelona database, the input dimensions were (8,51,9), where 8 represented the number of IMF components, and 51 and 9 represented the number of frequency points and time frames after STFT processing, respectively. The CNN utilized 16 filters with a kernel size of 3, a padding of 1, and AdaptiveAvgPool with output dimensions of 6, yielding output dimensions of (16,6,6). For the TUSZ database, the input dimensions were (56,51,9), where 56 represented the number of IMF components, and 51 and 9 represented the same meanings as above. The CNN utilized 128 filters while maintaining consistent values for other hyperparameters, resulting in output dimensions of (128,6,6). For all databases, BiGRU was configured with a hidden size of 512 and a layer number of 2, yielding output dimensions of (6,1024). Subsequently, the dimensions were reshaped to (48,128). A CLS Token was incorporated into the first dimension, expanding the tensor to dimensions of (49,128). Sinusoidal positional encoding was utilized for position encoding. Transformer was configured with a number of expected features of 128, a number of heads of 8, and a layer number of 3, yielding output dimensions of (49,128). The CLS Token was obtained to get output dimensions of (128). Final classification was accomplished through dense layers.

The experimental study was conducted on a single NVIDIA RTX 2060 GPU with 8 GB VRAM, utilizing Python 3.9 via the PyTorch 1.12.0 framework.

Accuracy (ACC), sensitivity (SEN), specificity (SPE), and weighted F1-score served as the primary evaluation metrics. To ensure robust performance validation, five-fold cross-validation was implemented to evaluate the algorithm’s effectiveness.

### 4.2. Experimental Results

To evaluate the impact of MVMD on classification performance, comparative experiments were conducted using EWT and VMD methods to decompose identical data as employed in this study. All methodologies maintained equivalent numbers of decomposition components. The EWT utilized the maxima method as the frequency band segmentation approach. VMD parameters remained consistent with those of MVMD. The classification experimental results of different decomposition methods are presented in Table 2. In this study, on the Bern–Barcelona database, we achieved 98.85%, 98.75%, and 98.95% for ACC, SEN, and SPE, respectively. On the TUSZ database, we obtained 96.17% for ACC and 0.962 for the weighted F1-score. These results demonstrate that the proposed MVMD decomposition method offered superior classification performance compared to EWT and VMD from the Bern–Barcelona and TUSZ databases.

For the Bern–Barcelona database, MVMD exhibited an enhancement in ACC of 4.38% and 1.9% over EWT and VMD, respectively, indicating an improved proportion of correct classifications. For the TUSZ database, MVMD surpassed EWT by 5.79% and VMD by 2.92% in ACC, demonstrating significant improvement in multi-class seizure type classification. The EWT and VMD methods yielded poorer classification results compared with MVMD. The comparatively inferior performance of EWT can be attributed to its limited adaptability in capturing transient signal components, where pathological epileptiform discharges (e.g., spike, sharp) may undergo erroneous segmentation into adjacent frequency bands, thereby compromising component integrity and decomposition performance. The enhanced classification performance of MVMD relative to VMD substantiates that inter-channel frequency consistency facilitates more effective feature representation, and the improved modal alignment across channels better accommodates the feature extraction capability of deep learning networks. The classification results demonstrated the effective ability of the MVMD decomposition method in classifying epileptic signals.

**Figure 5:**
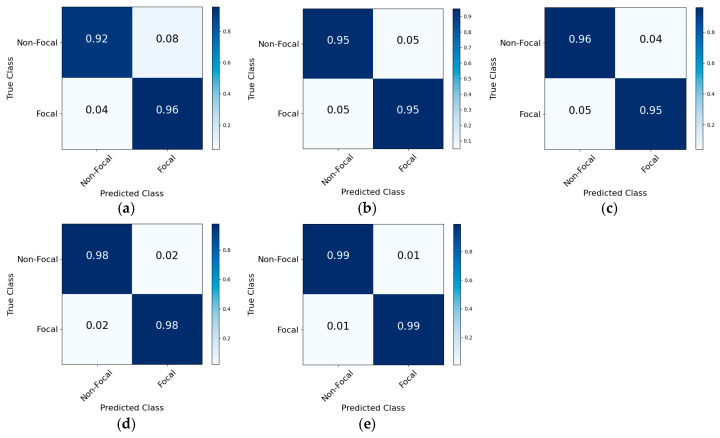
The normalized confusion matrix derived from ablation experiments using the Bern–Barcelona database. (**a**) Model without CNN component; (**b**) model without BiGRU module; (**c**) model eliminating Transformer component; (**d**) model excluding residual connections; (**e**) proposed framework.

**Figure 6:**
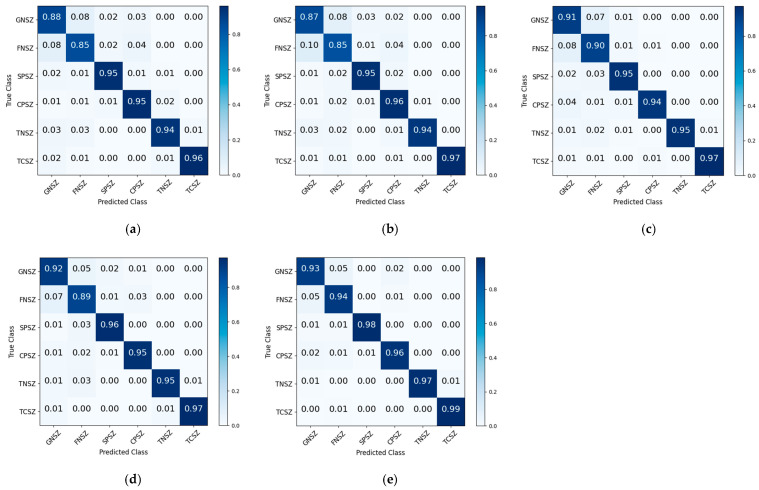
The normalized confusion matrix derived from ablation experiments using the TUSZ database. (**a**) Model without CNN component; (**b**) model without BiGRU module; (**c**) model eliminating Transformer component; (**d**) model excluding residual connections; (**e**) proposed framework.

To evaluate the contributions of individual components within the deep learning framework, ablation studies were performed to assess component performance. The evaluated model variants were configured as follows: (1) model without CNN component; (2) model without BiGRU module; (3) model eliminating the Transformer component; and (4) model excluding residual connections. The performance metrics derived from ablation experiments are summarized in Table 3. To provide explicit insights into the classification efficacy across types for different classification tasks, confusion matrices were employed to visualize classification results. Figure 5 presents the normalized confusion matrix obtained from ablation experiments using the Bern–Barcelona database. The normalized confusion matrix derived from ablation experiments using the TUSZ database is illustrated in Figure 6.

The experimental results on the Bern–Barcelona database revealed that the proposed framework achieved a correct classification rate of 0.99 for both non-focal and focal types. The absence of the CNN component led to reductions of 4.95%, 3.2%, and 6.7% in ACC, SEN, and SPE, respectively. The proportions of correctly classified non-focal and focal types decreased by 0.07 and 0.03, respectively. These substantial performance reductions indicated that the CNN component was effective in extracting local spatial features from spectrograms, thereby significantly reducing misclassification rates. When the BiGRU component was excluded, decreases of 4.05%, 3.75%, and 4.35% were observed in ACC, SEN, and SPE, respectively. Similarly, the removal of the Transformer component led to reductions of 3.05%, 3.25%, and 2.85% in these metrics. A comparative analysis revealed that BiGRU and Transformer components could obtain short-distance and long-distance temporal dependencies, respectively, with their combined utilization synergistically enhancing feature representation and classification efficacy. The elimination of residual connections induced marginal reductions in performance, suggesting that the implementation of cross-layer skip pathways facilitated the learning of complex mapping relationships, thereby affecting the classification performance. These findings demonstrated the complementary contributions of various components towards the classification performance.

The experimental results on the TUSZ database demonstrated that component exclusions led to significant reductions in classification accuracy and the weighted F1-score. The proposed framework achieved correct classification rates of 0.93, 0.94, 0.98, 0.96, 0.97, and 0.99 for GNSZ, FNSZ, SPSZ, CPSZ, TNSZ, and TCSZ types, respectively. In the absence of the CNN component, the proportions of correct classifications for GNSZ and FNSZ decreased by 0.05 and 0.09, respectively. Due to the lack of ability to obtain local spatial information, the proportions of GNSZ being misclassified as FNSZ and CPSZ reached 0.08 and 0.03, respectively, while the proportions of FNSZ being misclassified as GNSZ and CPSZ reached 0.08 and 0.04, respectively. In the absence of the BiGRU component, the proportions of correctly classified GNSZ, FNSZ, and SPSZ types decreased by 0.06, 0.09, and 0.03, respectively. When the Transformer component was removed, the proportions of correctly classified GNSZ and FNSZ types decreased by 0.02 and 0.04. These findings underscored that BiGRU and Transformer effectively extracted critical local and global temporal dependencies, respectively. A marginal reduction in performance was observed upon the removal of residual connections, suggesting their auxiliary function in facilitating complex mapping relationships. The ablation experiments conclusively demonstrated that each component played a crucial role in epileptic signal classification. The synergistic integration of these components enabled comprehensive feature extraction and improved classification efficacy, verifying the effectiveness and applicability of the framework.

## 5. Discussion

The TUSZ database, which contains detailed EEG recordings from epileptic patients, was utilized in this study to investigate the subject-independent experiment aimed at evaluating model generalizability across unseen patients. Subject-independent research poses significant challenges in improving model performance. This methodology ensures that no data from the same individual is shared between training and testing sets, thereby mitigating the risk of data leakage and enhancing generalization capability. Moreover, this approach aligns with clinical deployment requirements, as diagnostic systems need to maintain classification accuracy for new patients whose data were never involved in the training process, thereby ensuring the clinical applicability and reliability of the framework.

Based on the number of patients experiencing various seizure types, this study specifically conducted a subject-independent experiment for GNSZ, FNSZ, and CPSZ types. To facilitate this subject-independent analysis, three-fold cross-validation was implemented, splitting the patient data into three folds. No fold had patients in common with the others, ensuring that the data used for testing came from unseen patients.

The experimental results revealed an accuracy of 87.97% and a weighted F1-score of 0.884. A reduction in classification performance was observed compared to subject-dependent studies, indicating that the algorithm’s performance on unseen patients aligned more closely with real-world clinical scenarios. The maintained satisfactory classification performance demonstrated the model’s generalization capability across diverse patients, indicating the potential for clinical deployment. There are relatively limited subject-independent studies in the TUSZ database. Such investigations constitute a critical step toward practical clinical applications, as they assess the model’s capacity to maintain diagnostic performance on unseen patients and characterize the generalization ability.

Jia et al. used variable weight convolutional neural networks (VWCNNs) for epileptic seizure classification [41]. A weighted F1-score of 0.940 was achieved in the subject-dependent study. For subject-independent evaluations, the method was tested on 16 patients, with only 8 patients achieving correct classifications, resulting in an average accuracy of 54.17%. This method primarily focused on leveraging CNNs’ data processing capabilities, yet it lacked the integration of temporal information acquisition using deep learning methods as employed in this paper, leading to insufficient classification performance. The wavelet-based feature extraction, followed by a hybrid deep learning architecture combining a CNN and bidirectional long short-term memory (BiLSTM), was employed for classification [42]. Weighted F1-scores of 0.981 and 0.876 were obtained for subject-dependent and subject-independent studies, respectively. This approach involved manual feature extraction through wavelet-based signal decomposition, where six statistical features were derived from decomposed signals. Although superior classification performance was achieved, the reliance on empirical knowledge for feature engineering hindered the migration and application of algorithms in new scenarios. Albaqami et al. implemented DTCWT and LightGBM to achieve classification, attaining weighted F1-scores of 0.991 and 0.747 for subject-dependent and subject-independent evaluations, respectively [30]. The significant performance reductions in subject-independent experiments underscored the critical clinical implications for studies on unseen patients.

Table 4 summarizes a comparative analysis of the classification performance between the proposed framework and existing approaches on the Bern–Barcelona database. Sui et al. developed the time–frequency hybrid network (TFHybridNet), which employed STFT and a CNN [43]. Compared to this approach, our framework achieved improvements of 4.55% and 4.45% in ACC and SEN, respectively. These enhancements demonstrated superior performance in classifying focal epileptic signals through our framework that integrated spatial–temporal features, rather than relying solely on CNNs. Krishnan et al. implemented the Gramian angular summation field (GASF) approach to convert time series data into image representations, followed by classification using the RF classifier [44]. Our methodology demonstrated enhancements of 2.85%, 1.75%, and 3.95% in ACC, SEN, and SPE, respectively, relative to this technique, indicating more effective discrimination of focal epileptic signals. The combination of time–frequency signal analysis methods, including wavelet packet decomposition (WPD) and flexible analytic wavelet transform (FAWT), with entropy features has commonly been used for extracting features. These extracted features were trained using a least-squares support vector machine (LS-SVM) and an artificial neural network (ANN) to achieve classifications [45,46]. Our framework exhibited significant performance enhancements compared to these approaches, indicating that the integrated spatial–temporal deep learning network excelled in classification performance, thereby showing potential for precise identification of epileptogenic zones.

Table 5 summarizes a comparative analysis of the classification performance between the proposed framework and existing approaches on the TUSZ database. Huang et al. utilized multilevel wavelet decomposition combined with CNNs to extract joint features from temporal, frequency, and spatial domains [48]. Our framework achieved improvements of 1.7% and 0.018 in ACC and the F1-score, respectively, indicating the enhanced effectiveness in temporal information extraction through the integration of BiGRU and Transformer networks. The methods of the residual neural network (ResNet) and BiLSTM were employed for multi-class seizure type classification [49]. These methods extracted local spatial features and temporal dependencies through models. Our framework demonstrated enhancements of 1.14% and 0.012 in ACC and the weighted F1-score, respectively. This indicated the Transformer network’s capability to capture long-distance dependencies in signal sequences, thereby compensating for limitations in temporal feature extraction. Other advanced deep learning techniques, including VWCNNs, a graph-generative neural network (GGN), and a dynamic temporal graph convolutional network (DTGCN), have been utilized for multi-class seizure type classification [41,50,51]. Compared with these methods, our framework significantly improved classification performance, enabling more accurate classification of multi-class seizure types to assist epileptic physicians in making rapid and efficient clinical diagnoses.

The classification capability of the algorithm for epileptic signals was validated using two databases, thereby circumventing the limitations inherent in single-database evaluations, which may lead to insufficient generalization analysis. The superior classification performance from two databases demonstrated the algorithm’s robust feature extraction capability for diverse signal distributions and application environments, indicating its promising potential for diverse classification tasks. Furthermore, for the TUSZ database, subject-dependent and subject-independent scenarios were systematically evaluated to ensure clinical applicability. The algorithm exhibited exceptional classification accuracy on unseen patients, thereby confirming its strong capacity to generalize to new clinical cases and practical clinical utility.

This study has several limitations. The use of the signal decomposition technique and the deep learning framework results in a large amount of model parameter data, which limits hardware integration. Although this study employs two classification tasks to verify the performance of the method across tasks, it remains challenging for the method to handle more classification tasks.

There are several directions for future research. In the future, we will explore model compression and acceleration methods to achieve a lightweight architecture for the model, making it easier to integrate for the wearable detection device and real-time deployment. This will enable the implementation of remote monitoring platforms and long-term efficacy evaluation to optimize treatment plans. The transition of seizure types over time in epileptic patients may result in the coexistence of multiple seizure types within a single patient, necessitating timely and accurate clinical observation to facilitate comprehensive treatment planning by physicians. The failure to identify specific seizure types could lead to poor or even ineffective therapeutic effects. This clinical complexity presents significant challenges in epileptological management. With the continuous expansion and improvement of epileptic databases, future research will focus on the detection of seizure type changes for epileptic patients over time to adapt to complex clinical situations.

## 6. Conclusions

The proposed deep learning network framework was evaluated on two databases for epileptic signal classification. The method employs MVMD to achieve modal alignment of multi-channel epileptic signals, and then effectively classifies epileptic signals by capturing spatial information, along with local and global temporal information. These experimental results demonstrate robust generalization capabilities and show potential clinical utility in assisting neurologists with focal epileptic signal classification and multi-class seizure type classification.

## Figures and Tables

**Table 1:** A detailed description of the TUSZ database.

Seizure Type	Patient	Patient Used	Seizure	Seizure Used
GNSZ	81	36	583	337
FNSZ	150	63	1836	1078
ABSZ	12	-	99	-
SPSZ	3	1	52	8
CPSZ	41	11	367	114
TNSZ	3	2	62	61
TCSZ	14	3	48	10
MYSZ	2	-	3	-
Total	306	116	3050	1608

**Table 2:** The classification experimental results of different decomposition methods.

Decomposition Method	Bern–Barcelona			TUSZ	
	ACC (%)	SEN (%)	SPE (%)	ACC (%)	F1-Score
EWT	94.47	96.15	92.80	90.38	0.904
VMD	96.95	97.90	96.00	93.25	0.933
MVMD	98.85	98.75	98.95	96.17	0.962

**Table 3:** The performance metrics obtained from ablation experiments.

Model	Bern–Barcelona			TUSZ	
	ACC (%)	SEN (%)	SPE (%)	ACC (%)	F1-Score
Without CNN	93.90	95.55	92.25	92.17	0.922
Without BiGRU	94.80	95.00	94.60	92.38	0.924
Without Transformer	95.80	95.50	96.10	93.75	0.938
Without Residual Connection	97.72	97.65	97.80	94.13	0.942
Proposed Framework	98.85	98.75	98.95	96.17	0.962

**Table 4:** The classification results of different approaches on the Bern–Barcelona database.

Author(s)	Method	ACC (%)	SEN (%)	SPE (%)
Narin et al. [47]	CWT + Pre-trained CNN	92.27	92.40	92.93
Sui et al. [43]	TFHybridNet	94.30	94.30	-
You et al. [45]	FAWT + Entropies + LS-SVM	94.80	92.27	96.10
Sairamya et al. [46]	WPD + ANN	95.74	95.73	95.74
Krishnan et al. [44]	GASF + RF	96.00	97.00	95.00
Proposed	MVMD + Deep learning framework	98.85	98.75	98.95

**Table 5:** The classification results of different approaches on the TUSZ database.

Author(s)	Method	ACC (%)	F1-Score
Wu et al. [51]	DTGCN	-	0.759
Yan et al. [52]	Dynamic temporal–spatial graph attention network	89.20	0.893
Li et al. [50]	GGN with brain functional connectivity graphs	91.00	0.910
Hu et al. [53]	Iterative gated graph convolutional network	91.80	0.915
Jia et al. [41]	VWCNNs	-	0.940
Huang et al. [48]	Three-dimensional convolutional multiband model with attention mechanisms	94.47	0.944
Zhao et al. [49]	ResNet + BiLSTM	95.03	0.950
Proposed	MVMD + Deep learning framework	96.17	0.962

## Data Availability

The Bern–Barcelona database employed in this investigation is accessible via Reference [39]. The TUSZ database can be obtained from https://isip.piconepress.com/projects/nedc/html/tuh_eeg/#c_tusz (accessed on 20 June 2025).
